# Prognosis of second primary oral squamous cell carcinoma after hematologic malignancy: a retrospective cohort analysis

**DOI:** 10.3389/fonc.2025.1667226

**Published:** 2025-09-03

**Authors:** Huajiao Yu, Bo Li, Yu Huang, Xue Zhang, Hanchen Zhou, Zhien Feng, Zhengxue Han

**Affiliations:** ^1^ Department of Oral and Maxillofacial-Head and Neck Oncology, Beijing Stomatological Hospital, Capital Medical University, Beijing, China; ^2^ Department of Oral and Maxillofacial Surgery, Affiliated Hospital of Inner Mongolia Medical University, Hohhot, Inner Mongolia, China

**Keywords:** oral squamous cell carcinoma, hematologic malignancy, second primary malignancy, prognosis, surgical treatment

## Abstract

**Backgrounds:**

Prognosis and optimal management strategies of second primary oral squamous cell carcinoma (OSCC) following a history of hematologic malignancies (HM) remain uncertain. We investigated whether HM history affects OSCC outcomes or necessitates treatment modifications.

**Patients and methods:**

This retrospective cohort study included 2486 OSCC patients: 14 with OSCC as a second primary malignancy post-HM (SPM group) and 2472 with primary OSCC (non-SPM group). Using propensity score matching (PSM), we created two cohorts: 1:17 (13 SPM vs 232 non-SPM) and 1:3 (13 SPM vs 38 non-SPM). Outcomes were disease-free survival (DFS), overall survival (OS), and disease-specific survival (DSS). Survival differences were analyzed using log-rank tests. Multivariate Cox regression identified prognostic predictors.

**Results:**

No significant survival differences existed between SPM and non-SPM groups in either cohort (1:17: DFS 53.8% vs 68.9%, p=0.102; OS 69.2% vs 81.3%, p=0.170; DSS 69.2% vs 82.2%, p=0.147. 1:3: DFS 53.8% vs 63.2%, p=0.302; OS 69.2% vs 76.3%, p=0.532; DSS 69.2% vs 78.9%, p=0.430). Cox regression identified independent predictors: DFS: Age (p=0.001), T stage (p<0.001), N stage (p<0.001); OS and DSS: Age (p<0.001), T stage (p<0.001), N stage (p<0.001), pathological grade (p<0.001), prior HM was not an independent predictor.

**Conclusions:**

A history of HM does not independently predict the prognosis of second primary OSCC nor necessitate modifications to standard OSCC treatment.

## Introduction

Oral squamous cell carcinoma (OSCC) is one of the most prevalent head and neck malignancies, accounting for over 90% of oral cancers (1, 2). Despite advances in surgical and adjuvant therapies, its 5-year survival rate remains around 50% (3, 4). Tumor prognosis is influenced by a complex interplay of clinical, biological, and systemic factors (5–7). Underlying systemic diseases may profoundly impact cancer development, treatment efficacy, and outcomes (8–10).

Among systemic comorbidities, prior malignancies represent a distinct clinical entity (11, 12). In patients with dual primary tumors, prognosis may be affected by the biological characteristics of both malignancies, cumulative treatment-related toxicities, immune microenvironment remodeling (13), and molecular interactions. Patients with hematologic malignancies (HM), such as leukemia or lymphoma, face elevated risks of secondary solid tumors, including OSCC, primarily due to factors such as treatment-induced DNA damage (14) and immune microenvironment alterations (13). Notably, persistent immunosuppression (15) and chronic inflammation (16) in these individuals may promote a tumor-permissive microenvironment.

Although second primary malignancies (SPM) in cancer survivors, particularly those with prior HM, are increasingly studied, the independent prognostic impact of HM history on subsequent OSCC remains not well characterized. These patients often present compounded challenges from cumulative immunosuppression (15), impaired bone marrow function, and heightened susceptibility to therapy-related carcinogenesis (14).

This study aims to compare OSCC prognosis between patients with and without a history of hematologic malignancy, specifically examining whether well-controlled prior HM confers additional risk. We further seek to evaluate whether standard OSCC treatment protocols adequately meet the needs of this subgroup or require modification. Our findings are expected to inform clinical decision-making and guide treatment optimization for patients burdened by complex oncologic histories.

## Patients and methods

### Participants

This research was conducted in accordance with the World Medical Association’s Declaration of Helsinki (2002 revision). Ethical approval was obtained from the Institutional Review Board of the Beijing Stomatological Hospital, Capital Medical University (approval number: CMUSH-IRB-KJ-PJ-2022-38).

The study cohort comprised 2486 patients with OSCC who received treatment at the Department of Oral and Maxillofacial-Head and Neck Oncology, Beijing Stomatological Hospital, Capital Medical University from January 2000 to December 2021.

### Eligibility criteria

The tumors were reclassified in accordance with the 8th edition of the UICC/AJCC classification system, utilizing the initial clinical descriptions as the basis for restaging. The criteria for patient selection were as follows: (1) histopathological confirmation of malignant tumor, (2) the site of tumor is the oral and maxillofacial region. The exclusion criteria are as follows: (1) distant metastasis, (2) no surgical treatment received, (3) the lesion site is located in the oropharynx, jawbone and glands, (4) pathologically confirmed as non-squamous cell carcinoma.

### Treatments

All patients in this study received surgical treatment and adjuvant therapy according to different stages and the regimens recommended by the NCCN guidelines, repair and reconstruction was performed if necessary.

### Follow-up

All the enrolled patients received regular follow-ups. The follow-up for OSCC was conducted by the specialist of our center, while the follow-up for HM was carried out by the hematology physicians. All the follow-up results were recorded in the patients’ medical records, and the frequency for each patient is once every 3–6 months.

### Definitions

In this study, the SPM group was defined as patients whose first primary malignancy was a hematologic malignancy (HM) and whose second primary malignancy was OSCC. Patients presenting with OSCC as their first primary malignancy were classified as non-SPM. Disease-free survival (DFS), overall survival (OS) and disease-specific survival (DSS) were used as outcome variables to evaluate prognosis. The interval from the initial surgery to recurrence or death was used to calculate the DFS. OS was defined from the time of the initial surgery to death or the last follow-up. DSS was defined from the time of the initial surgery to the death cause from cancer or the last follow-up.

### Data analyses

Descriptive statistics were compiled to present the data in terms of frequencies and percentages. Comparative analysis of baseline demographic characteristics was conducted using the chi-square test for categorical variables. The Kaplan-Meier survival analysis was utilized to assess DFS, OS and DSS, with the log-rank test employed to evaluate the significance of differences between groups. Potential predictors were examined through cox proportional hazards regression analysis. All statistical tests were two-sided, with a significance level set at p<0.05. The statistical analyses were performed using SPSS software (version 21.0 for Windows) and R (version 4.3.1 for Windows).

To minimize selection bias, propensity score matching (PSM) was employed to balance the different groups in terms of baseline covariates, PSM could reduce confounding factors and improve comparability among different group, SPSS was used to implement PSM. We set the caliper value to 0.1 and the matching ratio of SPM group and non-SPM group to 1:17 (the sample size calculation is completed through PASS). Due to the sample size, a high proportion of matching may affect the results. Therefore, a matching ratio of 1:3 is also used for double testing of the results.

## Results

### Patients

**Figure 1:**
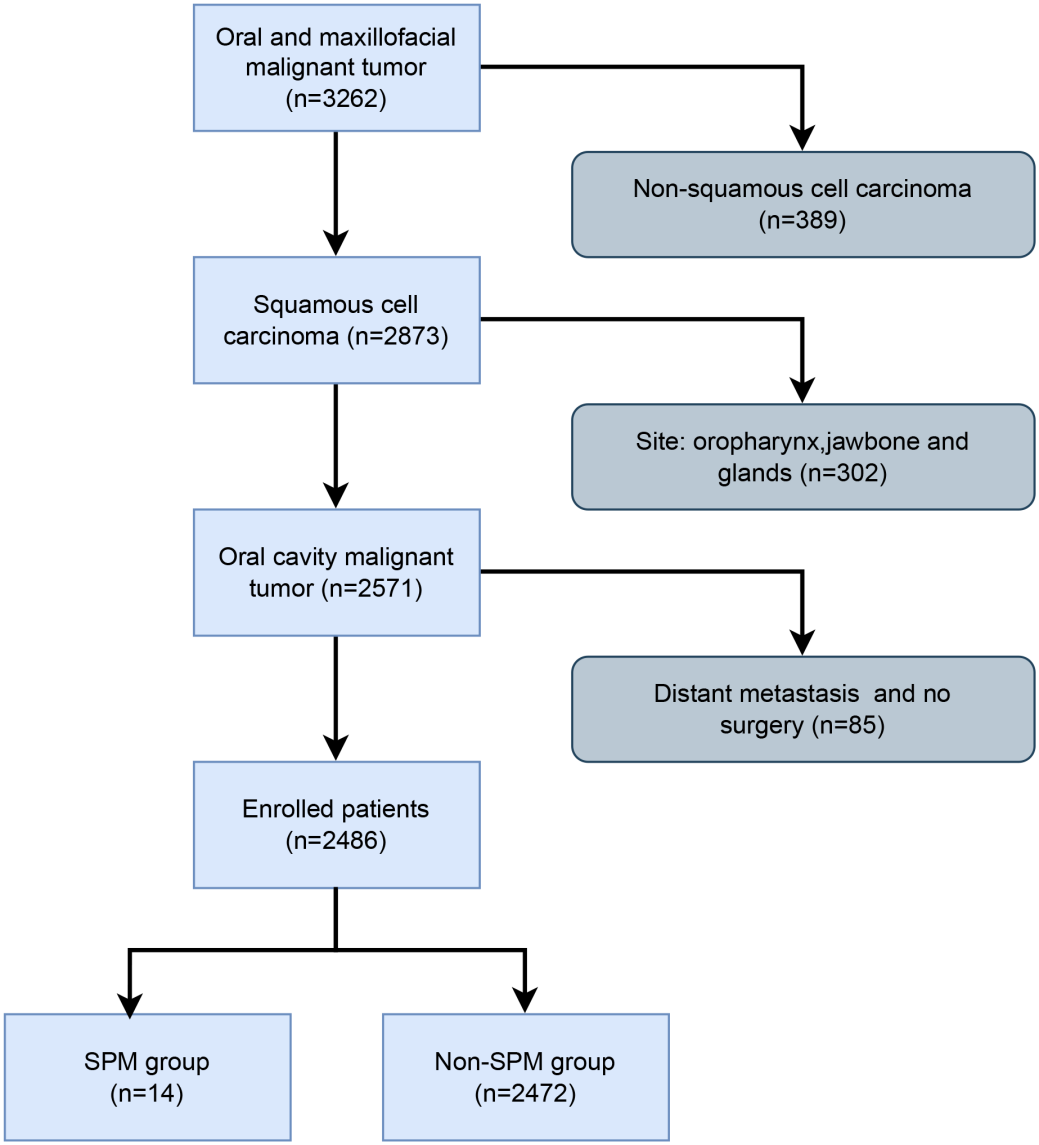
Flowchart for screening enrolled patients. Out of the 3262 patients, 2486 met the inclusion criteria. Among them, 14 were in the SPM group and 2472 were in the non-SPM group.

A total of 3262 patients with oral and maxillofacial malignant tumors who met the study time were included, through the inclusion and exclusion criteria for screening, a total of 2486 patients were finally included in this study, among which 14 cases (0.6%) were in the SPM group and 2472 cases (99.4%) were in the non-SPM group (**Figure 1**).

**Figure 2:**
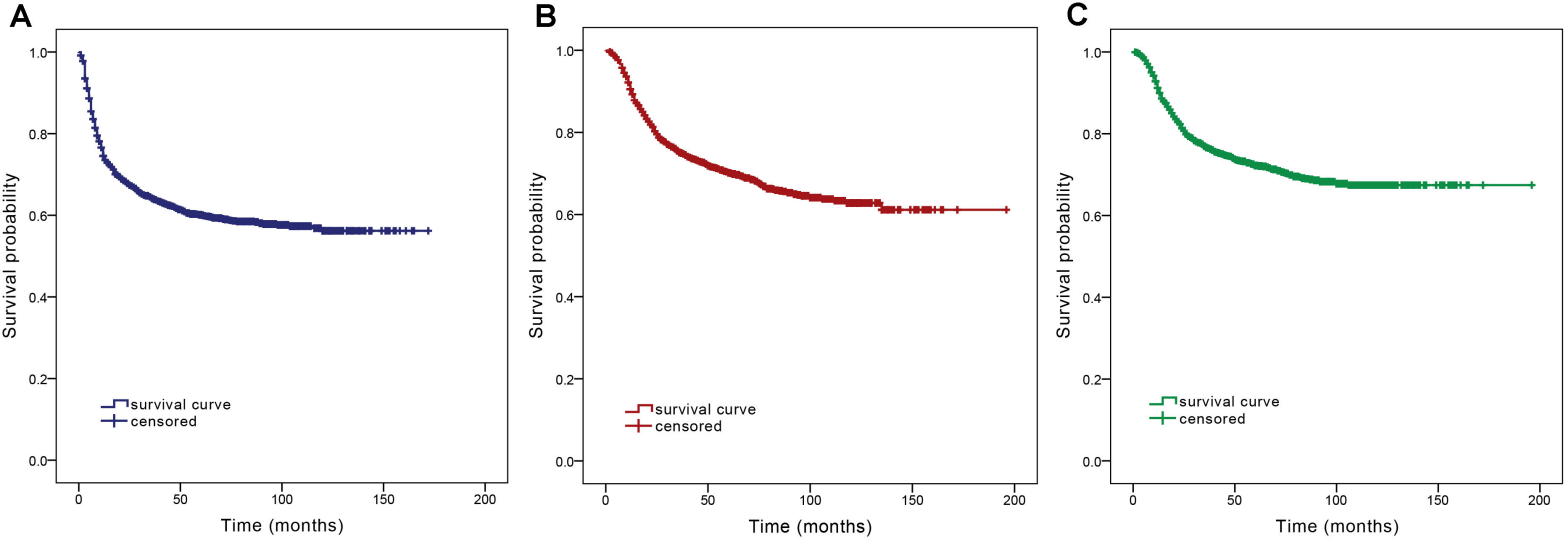
Survival curves of all enrolled patients: **(A)** DFS; **(B)** OS; **(C)** DSS.

Among the 2486 patients, 1428 were male (57.4%) and 1058 were female (42.6%). Mean age was 59.6 years, with a slight predominance of younger patients (1259, 50.6%) over older patients (1227, 49.4%). The tongue was the most common site in the overall situation, with a total of 1084 cases (43.6%), followed by the gums (684, 27.5%), buccal (418, 16.8%), floor of the mouth (237, 9.5%), and hard palate (63, 2.5%). The distribution of T stages is as follows: 21 cases (0.8%) in Tis stage, 563 cases (22.7%) in T1 stage, 854 cases (34.3%) in T2 stage, 313 cases (12.6%) in T3 stage, 663 cases (26.7%) in T4a stage, and 72 cases (2.9%) in T4b stage; Most patients were at the N0 stage (1656 cases, 66.6%) at the time of diagnosis, followed by the N2 stage (459 cases, 18.4%), the N1 stage (327 cases, 13.2%), and the N3 stage (44 cases, 1.8%). Among them, 1016 patients had a history of smoking (40.9%), and 1470 patients had no history of smoking (59.1%). 817 patients had a history of alcohol consumption, while 1669 patients had no history of alcohol consumption. The survival curve of the total queue is shown in **Figure 2**.

### Baseline characteristics and disease management in the SPM cohort

The SPM cohort (n=14) comprised 9 males and 5 females. Primary hematologic malignancies included leukemia (n=9) and lymphoma (n=5), the treatment of the first primary malignancy of patients in the SPM group includes bone marrow transplantation, immunosuppression, chemotherapy, radiotherapy, and surgical therapy, which are combined into different treatment regimens. Mean age at first primary malignancy diagnosis was 36.6 years and 50.1 years for SPM diagnosis. SPM sites included tongue (n=6), buccal (n=4), gum (n=3), and hard palate (n=1). Tumor staging revealed Tis (n=1), T1 (n=3), T2 (n=7), T3 (n=1), and T4a (n=2) disease and all patients underwent surgical treatment for OSCC, one patient received adjuvant radiotherapy. All patients had negative lymph nodes (N0 stage). Seven patients reported tobacco use history and four reported alcohol use history (**Table 1**).

**Table 1:** Basic information of patients in the SPM group.

Number	Gender	FM	Age (FM)	Treatment (FM)	Age (SM)	Site (SM)	T stage	N stage	Tobacco/Alcohol
Patient 1	Male	Leukemia	35	BMT+IST+CT	50	Tongue	T3	N0	Yes/No
Patient 2	Male	Leukemia	40	BMT+CT	48	Tongue	T2	N0	Yes/Yes
Patient 3	Male	Leukemia	33	BMT+CT	41	Buccal	T2	N0	No/No
Patient 4	Male	Leukemia	34	BMT+CT	43	Buccal	T2	N0	No/No
Patient 5	Female	Leukemia	14	BMT+CT	16	Buccal	T1	N0	No/No
Patient 6	Female	Leukemia	18	BMT+CT	27	Tongue	T2	N0	No/No
Patient 7	Male	Leukemia	33	BMT+CT	47	Tongue	T2	N0	Yes/Yes
Patient 8	Male	Leukemia	38	BMT+IST+CT	50	Buccal	Tis	N0	Yes/Yes
Patient 9	Male	Lymphoma	59	CT	69	Hard Palate	T2	N0	Yes/No
Patient 10	Male	Lymphoma	53	CT+RT	63	Gum	T4a	N0	Yes/Yes
Patient 11	Female	Lymphoma	70	CT+RT	76	Tongue	T1	N0	No/No
Patient 12	Male	Lymphoma	28	ST+CT	39	Gum	T4a	N0	Yes/No
Patient 13	Female	Lymphoma	8	ST+CT+RT	66	Tongue	T1	N0	No/No
Patient 14	Female	Leukemia	50	CT	66	Gum	T2	N0	No/No

FM, first malignance; SM, second malignance; BMT, bone marrow transplantation; IST, immunosuppressive therapy; CT, chemotherapy; RT, radiotherapy; ST, surgical treatment.

### Baseline characteristics after propensity score matching

PSM was performed using 1:17 and 1:3 ratio to screen out comparable datasets. Ultimately, in 1:17 matching a total of 232 patients were included in the matching cohort, with 13 cases in the SPM group and 219 cases in the non-SPM group, the baseline data are shown in **Table 2**. In another group of pairs matched at a ratio of 1:3, a total of 51 patients were included, including 13 in the SPM group and 38 in the non-SPM group (**Table 3**).

**Table 2:** Patient characteristics after propensity score matching by 1:17.

Variable	Non-SPM group (n=219)	SPM group (n=13)	p-value	SMD
Age			0.681	0.077
≤60	122	8		
>60	97	5		
Gender			0.410	0.004
Male	109	8		
Female	110	5		
Site			0.778	0.123
Tongue	94	6		
Gum	75	3		
Buccal	42	3		
Hard palate	8	1		
T stage			0.959	0.022
T1	51	3		
T2	103	7		
T3	21	1		
T4	44	2		
Pathological grade			0.507	0.086
Well	138	7		
moderately	81	6		
Tobacco use			0.382	0.033
Yes	75	6		
No	144	7		
Alcohol use			0.813	0.063
Yes	57	3		
No	162	10		

**Table 3:** Patient characteristics after propensity score matching by 1:3.

Variable	Non-SPM group (n=38)	SPM group (n=13)	p-value	SMD
Age			0.577	0.155
≤60	20	8		
>60	18	5		
Gender			0.818	0.103
Male	22	8		
Female	16	5		
Site			0.843	0.120
Tongue	13	6		
Gum	11	3		
Buccal	12	3		
Hard palate	2	1		
T stage			0.985	<0.100
T1	9	3		
T2	20	7		
T3	2	1		
T4	7	2		
Pathological grade			0.799	0.100
Well	22	7		
moderately	16	6		
Tobacco use			0.811	<0.100
Yes	19	6		
No	19	7		
Alcohol use			0.878	<0.100
Yes	8	3		
No	30	10		

### Comparable prognosis in SPM and non-SPM groups

**Figure 3:**
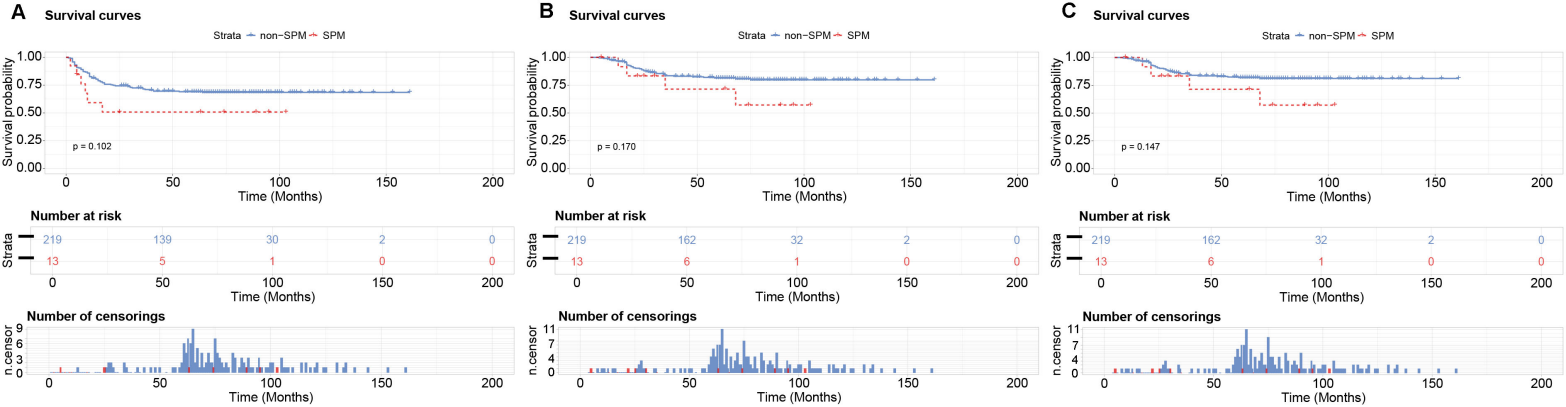
Kaplan-Meier survival analysis of the PSM cohort (1:17 matching ratio). No statistically significant differences were observed between SPM group and non-SPM group. **(A)** DFS: 53.8% vs. 68.9% (p=0.102); **(B)** OS: 69.2% vs. 81.3% (p=0.170); **(C)** DSS: 69.2% vs. 82.2% (p=0.147).

**Figure 4:**
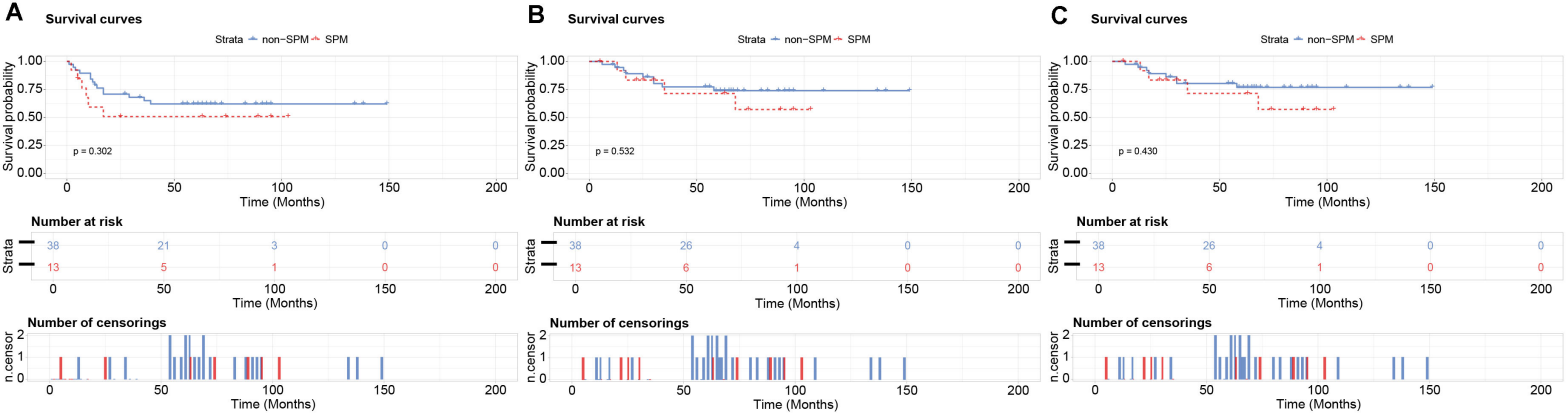
Kaplan-Meier survival analysis of the PSM cohort (1:3 matching ratio). No statistically significant differences were observed between SPM group and non-SPM group. **(A)** DFS: 53.8% vs. 63.2% (p=0. 0.302); **(B)** OS: 69.2% vs. 76.3% (p=0.532); **(C)** DSS: 69.2% vs. 78.9% (p=0.430).

Kaplan-Meier analysis revealed no significant prognostic differences between groups. In the 1:17 matched cohort, DFS (53.8% vs. 68.9%, p=0.102), OS (69.2% vs. 81.3%, p=0.170) and DSS (69.2% vs. 82.2%, p=0.147) did not show significant statistical differences in SPM group and non-SPM group (**Figure 3**). The similar results were obtained in 1:3 matching cohort, which indicated that DFS (53.8% vs. 63.2%, p=0.302), OS (69.2% vs. 76.3%, p=0.532) and DSS (69.2% vs. 78.9%, p=0.430), no significant statistical difference was observed between SPM group and non-SPM group (**Figure 4**). These results suggest that among patients with prior hematologic malignancies, OSCC as a second primary malignancy confers no significantly different prognosis compared to OSCC as a first primary malignancy.

### Independent predictors were screened through Cox regression analysis

Multivariable Cox proportional hazards regression was performed to identify independent prognostic factors across survival endpoints. The analysis established age as a consistent predictor for all outcomes, with significantly increased risk observed for DFS (p=0.001), OS (p<0.001), and DSS (p<0.001). Similarly, advanced T stage and N stage demonstrated strong independent associations with reduced survival across all three endpoints (p<0.001 for each). Notably, pathological grade emerged as a powerful independent determinant for both OS (p<0.001) and DSS (p<0.001), suggesting its differential impact on survival outcomes (**Supplementary Table 1**).

## Discussion

The prognosis of OSCC is multifactorial, with systemic health status representing a key determinant. Among systemic comorbidities, a history of prior malignancy—particularly HM—constitutes a distinct clinical entity.

HM may influence the development and progression of SPMs. First, HM inherently induce systemic immune impairment, including T-cell dysfunction, diminished NK-cell activity, and compromised humoral immunity (17–20), this significantly weakens immune surveillance against solid tumors (21, 22). Second, HM treatments further impact SPM risk: conventional radiotherapy and chemotherapy regimens exacerbate immune injury, causing persistent lymphocyte subset imbalances. These therapies represent a double-edged sword—while targeting malignant cells, they simultaneously inflict DNA damage on healthy tissues in a dose-dependent manner (23–25). Additionally, the risk of SPM in HM patients after CART treatment should also be vigilant (26). Consequently, immune reconstitution failure may not only enhance tumor aggressiveness in second primary cancers but also diminish responsiveness to immunotherapy. Collectively, systemic immune dysregulation and chronic inflammation establish a tumor-promoting milieu.

HM survivors frequently exhibit persistent antigenic stimulation and inflammatory microenvironments that facilitate OSCC pathogenesis through multiple molecular pathways. The neutrophil-to-lymphocyte ratio, a validated systemic inflammation biomarker, demonstrates significant correlation with OSCC prognosis (27, 28). In HM survivors, this pro-inflammatory state often persists due to underlying disease and prior therapies.

Importantly, the prognosis of HM patients developing into SPM is still not completely clear, and the reasons may be multi-faceted. On the one hand, the causes of SPM in HM patients are very complex. It may be related to the impact of the HM disease itself on the overall condition, or it may be related to the complex treatment plan of HM. On the other hand, such cases are usually few in number and it is difficult to complete effective statistical analysis.

Our large-sample cohort study specifically examined OSCC outcomes in patients with prior HM. While concerns historically existed regarding treatment planning for this population—given potential HM-related comorbidities and uncertain prognosis—our exploratory analysis provides clinical guidance. Current findings indicate that a history of well-controlled HM does not constitute an independent high-risk prognostic factor for OSCC. Thus, patients developing OSCC as SPM after HM should be considered for conventional OSCC therapy.

Study limitations include the relatively small SPM subgroup size, which constrains statistical power. Nevertheless, large-scale cohort analysis remains the optimal feasible approach. Future work will expand recruitment to strengthen these findings.

## Conclusions

Following conventional OSCC therapy, no statistically significant difference in prognosis was observed between patients with OSCC as a second primary malignancy after HM and those with primary OSCC, indicating that treatment modification appears unnecessary for these patients.

## Data Availability

The original contributions presented in the study are included in the article/**Supplementary Material**. Further inquiries can be directed to the corresponding authors.
