# Supplementary material for: Prognosis of second primary oral squamous cell carcinoma after hematologic malignancy: a retrospective cohort analysis
Source: Front Oncol. 2025 Sep 3;15:1667226. doi: 10.3389/fonc.2025.1667226 (PMC12442044; doi:10.3389/fonc.2025.1667226)
Supplement: Supplementary file 1 [file Table1.docx]

Supplementary Table 1. The Cox risk regression models of the cohort.

| Variable | Hazard ratio | 95% CI | p-value |
| --- | --- | --- | --- |
| **Univariate analysis for DFS** |  |  |  |
| Age (>60 vs. ≤60) | 1.150 | 1.008 to 1.312 | 0.038 |
| gender (female vs. male) | 0.945 | 0.826 to 1.082 | 0.413 |
| Site |  |  | 0.332 |
| Tongue | Ref |  |  |
| Gum | 1.015 | 0.857 to 1.204 |  |
| Buccal | 1.100 | 0.906 to 1.335 |  |
| Floor of mouth | 1.188 | 0.938 to 1.504 |  |
| Hard palate | 0.532 | 0.220 to 1.287 |  |
| T stage |  |  | <0.001 |
| Tis | Ref |  |  |
| T1 | 3.465 | 1.104 to 10.871 |  |
| T2 | 4.708 | 1.510 to 14.681 |  |
| T3 | 5.962 | 1.894 to 18.763 |  |
| T4a | 6.567 | 2.105 to 20.486 |  |
| T4b | 9.225 | 2.833 to 30.038 |  |
| N stage |  |  | <0.001 |
| N0 | Ref |  |  |
| N1 | 1.530 | 1.268 to 1.846 |  |
| N2 | 2.539 | 2.179 to 2.957 |  |
| N3 | 4.807 | 2.770 to 8.343 |  |
| Pathological grade |  |  | 0.773 |
| Well | Ref |  |  |
| Moderate | 0.958 | 0.831 to 1.104 |  |
| Poor | 1.044 | 0.771 to 1.415 |  |
| Tobacco use (yes vs. no) | 1.116 | 0.978 to 1.275 | 0.104 |
| Alcohol use (yes vs. no) | 1.117 | 0.973 to 1.282 | 0.117 |
| Group (SPM vs. non-SPM) | 1.250 | 0.560 to 2.790 | 0.586 |
| **Multivariate analysis for DFS** |  |  |  |
| Age (>60 vs. ≤60) | 1.245 | 1.090 to 1.422 | 0.001 |
| T stage |  |  | <0.001 |
| Tis | Ref |  |  |
| T1 | 3.874 | 1.226 to 12.075 |  |
| T2 | 4.632 | 1.485 to 14.450 |  |
| T3 | 5.182 | 1.645 to 16.326 |  |
| T4a | 5.466 | 1.750 to 17.072 |  |
| T4b | 7.361 | 2.258 to 23.999 |  |
| N stage |  |  | <0.001 |
| N0 | Ref |  |  |
| N1 | 1.458 | 1.205 to 1.765 |  |
| N2 | 2.326 | 1.979 to 2.734 |  |
| N3 | 4.136 | 2.362 to 7.242 |  |
| **Univariate analysis for OS** |  |  |  |
| Age (>60 vs. ≤60) | 1.272 | 1.098 to 1.473 | 0.001 |
| gender (female vs. male) | 0.758 | 0.650 to 0.883 | <0.001 |
| Site |  |  | 0.015 |
| Tongue | Ref |  |  |
| Gum | 1.168 | 0.970 to 1.407 |  |
| Buccal | 1.066 | 0.851 to 1.335 |  |
| Floor of mouth | 1.410 | 1.093 to 1.820 |  |
| Hard palate | 1.477 | 0.964 to 2.263 |  |
| T stage |  |  | <0.001 |
| Tis | Ref |  |  |
| T1 | 5.157 | 0.718 to 37.018 |  |
| T2 | 9.123 | 1.280 to 65.045 |  |
| T3 | 13.085 | 1.825 to 93.812 |  |
| T4a | 15.629 | 2.193 to 111.379 |  |
| T4b | 20.748 | 2.826 to 152.318 |  |
| N stage |  |  | <0.001 |
| N0 | Ref |  |  |
| N1 | 2.139 | 1.746 to 2.621 |  |
| N2 | 3.824 | 3.239 to 4.514 |  |
| N3 | 7.688 | 4.413 to 13.391 |  |
| Pathological grade |  |  | <0.001 |
| Well | Ref |  |  |
| Moderate | 1.837 | 1.574 to 2.145 |  |
| Poor | 2.329 | 1.766 to 3.071 |  |
| Tobacco use (yes vs. no) | 1.334 | 1.152 to 1.545 | <0.001 |
| Alcohol use (yes vs. no) | 1.248 | 1.073 to 1.452 | 0.004 |
| Group (SPM vs. non-SPM) | 0.960 | 0.359 to 2.565 | 0.935 |
| **Multivariate analysis for OS** |  |  |  |
| Age (>60 vs. ≤60) | 1.433 | 1.236 to 1.661 | <0.001 |
| T stage |  |  | <0.001 |
| Tis | Ref |  |  |
| T1 | 6.019 | 0.838 to 43.243 |  |
| T2 | 8.775 | 1.230 to 62.616 |  |
| T3 | 10.980 | 1.530 to 78.811 |  |
| T4a | 11.659 | 1.634 to 83.186 |  |
| T4b | 15.969 | 2.173 to 117.331 |  |
| N stage |  |  | <0.001 |
| N0 | Ref |  |  |
| N1 | 1.844 | 1.497 to 2.271 |  |
| N2 | 2.902 | 2.421 to 3.479 |  |
| N3 | 6.886 | 3.905 to 12.144 |  |
| Pathological grade |  |  | <0.001 |
| Well | Ref |  |  |
| Moderate | 1.453 | 1.235 to 1.710 |  |
| Poor | 1.599 | 1.202 to 2.128 |  |
| **Univariate analysis for DSS** |  |  |  |
| Age (>60 vs. ≤60) | 1.192 | 1.022 to 1.390 | 0.026 |
| gender (female vs. male) | 0.781 | 0.666 to 0.917 | 0.003 |
| Site |  |  | 0.019 |
| Tongue | Ref |  |  |
| Gum | 1.170 | 0.962 to 1.421 |  |
| Buccal | 1.005 | 0.790 to 1.278 |  |
| Floor of mouth | 1.417 | 1.086 to 1.848 |  |
| Hard palate | 1.548 | 1.000 to 2.397 |  |
| T stage |  |  | <0.001 |
| Tis | Ref |  |  |
| T1 | 4.242 | 0.589 to 30.533 |  |
| T2 | 8.682 | 1.217 to 61.918 |  |
| T3 | 12.677 | 1.767 to 90.944 |  |
| T4a | 14.889 | 2.089 to 106.134 |  |
| T4b | 18.298 | 2.479 to 135.047 |  |
| N stage |  |  | <0.001 |
| N0 | Ref |  |  |
| N1 | 2.243 | 1.813 to 2.776 |  |
| N2 | 4.041 | 3.395 to 4.809 |  |
| N3 | 8.697 | 4.985 to 15.172 |  |
| Pathological grade |  |  | <0.001 |
| Well | Ref |  |  |
| Moderate | 1.900 | 1.614 to 2.236 |  |
| Poor | 2.353 | 1.757 to 3.151 |  |
| Tobacco use (yes vs. no) | 1.354 | 1.161 to 1.580 | <0.001 |
| Alcohol use (yes vs. no) | 1.256 | 1.072 to 1.472 | 0.005 |
| Group (SPM vs. non-SPM) | 1.047 | 0.392 to 2.798 | 0.927 |
| **Multivariate analysis for DSS** |  |  |  |
| Age (>60 vs. ≤60) | 1.354 | 1.160 to 1.581 | <0.001 |
| T stage |  |  | <0.001 |
| Tis | Ref |  |  |
| T1 | 4.956 | 0.688 to 35.715 |  |
| T2 | 8.262 | 1.157 to 58.975 |  |
| T3 | 10.442 | 1.454 to 75.006 |  |
| T4a | 10.835 | 1.518 to 77.338 |  |
| T4b | 13.949 | 1.888 to 103.045 |  |
| N stage |  |  | <0.001 |
| N0 | Ref |  |  |
| N1 | 1.885 | 1.515 to 2.345 |  |
| N2 | 2.988 | 2.472 to 3.612 |  |
| N3 | 7.630 | 4.315 to 13.491 |  |
| Pathological grade |  |  | <0.001 |
| Well | Ref |  |  |
| Moderate | 1.478 | 1.245 to 1.755 |  |
| Poor | 1.603 | 1.186 to 2.166 |  |
